# Exposure Pattern Determines Methamphetamine‐Induced Right Ventricular Dysfunction and Vascular Remodeling

**DOI:** 10.1002/cph4.70191

**Published:** 2026-06-04

**Authors:** Ashok Kumar, Aatish Mahajan, Balaji Krishnamachary, Ling Chen, Navneet K. Dhillon

**Affiliations:** ^1^ Division of Pulmonary, Critical Care and Sleep Medicine, Department of Internal Medicine University of Kansas Medical Center Kansas City Kansas USA

## Abstract

Methamphetamine (MA) use is a recognized risk factor for drug‐associated pulmonary arterial hypertension (PAH), yet mechanisms driving methamphetamine‐associated PAH (MA‐PAH) remain unclear due to the lack of reproducible animal models demonstrating hemodynamic changes. This study aimed to develop a rat model of MA‐PAH that reflects human patterns of MA abuse. Male Wistar rats received methamphetamine (MA, 5 mg/kg, i.p.) for 8 weeks using either a chronic daily regimen or a binge‐and‐crash regimen. Chronic MA administration resulted in a significant reduction in mean arterial pressure and an increase in RV systolic pressure, while ejection fraction and the Fulton index remained unchanged. In contrast, binge‐crash MA exposure led to significant RV dilation, reduced RV ejection fraction, elevated RV systolic pressure, and increased RV hypertrophy. Both regimens induced pulmonary arterial medial thickening driven by smooth muscle hyperplasia; however, the binge‐and‐crash model exhibited more severe remodeling, including enhanced distal muscularization, sporadic endothelial proliferation, and vascular rarefaction. HMGB1 levels were significantly elevated in plasma and pulmonary microvascular endothelial cells isolated from binge‐crash MA rats. In vitro, MA treatment induced HMGB1 release from endothelial cells through sigma‐1 receptor signaling. Conditioned media from MA‐treated endothelial cells stimulated smooth muscle cell proliferation, an effect abolished by HMGB1 neutralization. In conclusion, these findings demonstrate that binge‐and‐crash MA exposure produces a robust and clinically relevant PAH phenotype and identify HMGB1 as a potential mechanistic contributor linking MA exposure patterns to endothelial‐smooth muscle interactions in PAH pathogenesis.

## Graphical abstract

Chronic methamphetamine (MA) exposure caused mild, compensated pulmonary hypertension, whereas binge‐and‐crash exposure produced severe PAH with right‐ventricular dysfunction and marked vascular remodeling. Binge‐crash rats showed elevated plasma HMGB1, while in vitro methamphetamine triggered endothelial HMGB1 release that promoted smooth‐muscle proliferation, implicating HMGB1‐dependent endothelial‐smooth muscle signaling in PAH progression. MAP, systemic mean arterial pressure; RV EDA, right ventricular end diastolic area; RV EF, right ventricular ejection fraction; RVSP, right ventricular systolic pressure.

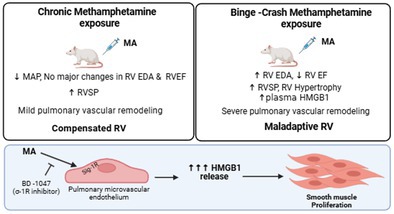

## 1 Introduction

Methamphetamine (MA) is a profoundly addictive substance that has seen a substantial rise in use in the United States. Between 2015 and 2019, overall MA use increased by 43%, while non‐injection methamphetamine use disorder rose by an alarming 105% during the same period (Han et al. 2021). MA has only recently been recognized as an independent risk factor for pulmonary arterial hypertension (PAH). Studies in idiopathic PAH (IPAH) patients have reported a ten‐fold higher prevalence of stimulant use compared with PAH patients who have established risk factors (Humbert et al. 2022; Chin et al. 2006). A nationwide analysis of U.S. inpatient records from 2008 to 2020 further demonstrated a steep rise in PAH‐related hospitalizations among MA users. Affected individuals were more frequently male, middle‐aged, and from lower‐income communities, with the highest burden observed in the Western and Southern United States (Husein et al. 2024). Multiple clinical studies also show that individuals with methamphetamine‐associated PAH (MA‐PAH) have worse right heart function, functional class, and overall prognosis compared with patients with other forms of pulmonary hypertension (Zamanian et al. 2018; Kolaitis et al. 2021; Ramirez 3rd et al. 2018; Charoenpong et al. 2023).

Given the rising prevalence of MA use and its strong association with PAH, understanding the underlying mechanisms is critical. Several studies have attempted to investigate the mechanisms driving MA‐induced pulmonary vascular remodeling. Proposed contributors include reduced carboxylesterase‐1 activity (Orcholski et al. 2017), increased oxidative stress (McDonnell‐Dowling and Kelly 2017; Liang et al. 2020), and dysregulation of serotonin pathways (Wang et al. 2018; Chen et al. 2017). However, the precise causal mechanism leading to pulmonary vascular remodeling in MA‐PAH remains unclear. One major limitation has been the lack of preclinical animal models that demonstrate reproducible hemodynamic changes, including right ventricular systolic pressure (RVSP) in response to MA exposure. Several prior attempts to induce PAH in rodents using MA, with or without hypoxia, failed to show RVSP elevation (Liang et al. 2020; Labazi et al. 2021; Bai et al. 2017; Wang et al. 2017a). A contributing factor may be the markedly different pharmacokinetics of MA between species (Melega et al. 1995; Riviere et al. 1999) (Cook et al. 1992; Harris et al. 2003). This discrepancy may have led to suboptimal dosing strategies in earlier models.

In humans, MA is commonly used in a binge‐and‐crash pattern. Although blood MA levels remain elevated for several hours, the euphoric effects dissipate rapidly, leading individuals to repeatedly consume the drug to maintain the high (Simon et al. 2002) (Cho and Melega 2002; Simon et al. 2000; Volkow et al. 2010) This cycle of binge consumption followed by a crash characterized by withdrawal and depressive symptoms drives long‐term, repetitive MA exposure (Cheng et al. 2010; Cruickshank and Dyer 2009).

HMGB1 is a well‐established damage‐associated molecular pattern (DAMP) that contributes to pulmonary arterial hypertension (PAH) pathogenesis. It is released during cellular stress and activates receptors such as RAGE and TLRs, amplifying inflammation (Harris et al. 2012). In PAH, HMGB1 promotes pulmonary vascular remodeling by driving smooth muscle cell proliferation and endothelial dysfunction. Elevated HMGB1 levels have also been associated with disease severity, highlighting its relevance as one of the mechanistic mediators (Bauer et al. 2013; Huang et al. 2016).

In this study, we developed a preclinical rat model of MA‐PAH that more accurately mimics human patterns of MA abuse. We observed significant hemodynamic alterations and pulmonary vascular remodeling following binge‐crash MA exposure. We further identified a strong association between extracellular high‐mobility group box 1 (HMGB1), a classic damage‐associated molecular pattern (DAMP), and enhanced pulmonary vascular remodeling characteristic of PAH (Zemskova et al. 2020).

## 2 Methods

### 2.1 Animals and Drug Dosing

Male Wistar rats (*n* = 6–8/group) weighing 220–280 g (ENVIGO, Indianapolis, IN, USA) received 5 mg/kg of (+) methamphetamine (Sigma Aldrich, USA), dissolved in saline intraperitoneally (i.p.). For the chronic MA regimen, rats were administered MA four times daily, at 6‐h intervals, 7 days a week, for 8 weeks. For the binge and crash regimen, rats received MA four times daily at 2‐h intervals for 5 consecutive days, followed by 2 drug‐free days each week, repeated for 8 weeks. For the placebo group, rats received saline solution four times daily for 8 weeks. All animals were housed at the University of Kansas Medical Center in strict accordance with the NIH and Institutional Animal Care and Use Committee guidelines. Rats were weighed weekly, housed individually under a 12 h/12 h light–dark cycle, and had ad‐libitum access to food and water.

### 2.2 Echocardiography, Hemodynamics, and Tissue Harvesting

Transthoracic echocardiography was performed on anesthetized rats (Ketamine/Xylazine, 50 mg/kg: 10 mg/kg, i.p.), on Day 0 and Day 56 to measure the end‐diastolic area (EDA) and ejection fraction (EF). At the end of treatment on day 56, echocardiography was followed by hemodynamic assessment. A midline incision was made to access the left carotid artery and right jugular vein. A polyethylene catheter (PE 50; inner diameter 0.58 mm, outer diameter 0.965 mm; BD Intramedic TM, Clay Adams) was advanced into the aortic arch via the left carotid artery, and a Renasil silicone catheter (inner diameter 0.25 mm, outer diameter 0.47 mm) was advanced into the RV through the right jugular vein. Proper catheter placement was confirmed via pressure waveforms. Mean arterial pressure (MAP) and RV systolic pressure (RVSP) were recorded on the PowerLab Data Acquisition System (ADInstruments Inc., Colorado Springs, CO, USA) and analyzed with the LabChart System (AD Instruments Inc., Version 8.0 Pro, Colorado Springs, CO, USA). Rats were euthanized by exsanguination, and the pulmonary artery, lungs, and heart were harvested. RV hypertrophy (RVH) was assessed using the Fulton index (RV/LV + septum). Portions of RV and LV + septum tissues were fixed in 4% paraformaldehyde for histology, and the remaining tissue was snap frozen. Lung lobes were similarly processed, with the left lobe snap‐frozen for endothelial cell isolation, RNA and protein analysis, and the right lobe was fixed for histological evaluation.

### 2.3 Immunohistochemistry

Paraformaldehyde‐fixed lung tissues were paraffin‐embedded and sectioned. Immunofluorescence staining for α‐smooth muscle actin (ɑ‐SMA; Abcam, USA) and von Willebrand factor (vWF; Agilent DAKO, USA) was performed with DAPI used for nuclear staining. Images were acquired using a Nikon 80i fluorescence microscope. For the quantification of vessel wall thickness, ɑ‐SMA‐stained lung sections were imaged under bright field mode with a Nikon Eclipse Ni‐U Tiling microscope. Subsequently, pulmonary vessels were categorized based on diameter (> 100 μm, 50–100 μm, < 50 μm). Wall thickness (%) was calculated as follows: Outer Area‐Inner Area/outer area × 100. Approximately 8–10 vessels per size group were quantified per rat.

### 2.4 Cell‐Culture

Human pulmonary microvascular endothelial cells (HPMECs) were cultured in 6‐well plates, serum starved, and treated with or without sigma‐1 receptor inhibitor BD‐1047 (10 nM) 30 min before MA (100 μM) exposure. Supernatants were collected at 48 h and 96 h and analyzed for HMGB1 by ELISA. Primary human pulmonary arterial smooth muscle cells (HPASMC) were grown in smooth muscle cell media (SMCM; ScienCell, USA) with growth factors, 2% fetal bovine serum, until 70% confluence in 96‐well plates and then serum‐deprived for 48 h. HPASMCs were then treated with HPMEC supernatants, and proliferation was assessed at 48 and 96 h using the CyQUANT Cell Proliferation Assay Kit (Invitrogen, C7026). In parallel experiments, HPASMCs were treated with MA‐conditioned or control HPMEC supernatants in the presence of anti‐HMGB1 neutralizing antibody (10 μg; ARG66714, SQab20175) or IgM isotype control. Rat pulmonary arterial smooth muscle cells (RPASMCs) were isolated as previously described (Dalvi et al. 2016) RPASMCs proliferation was assessed on days 2, 4, and 6 using MTS Cell Proliferation (Promega, USA) assay.

### 2.5 Measurement of HMGB1

HMGB1 levels in rat plasma and HPMEC supernatants were quantified using ELISA according to the manufacturer's instructions (Novus, USA). Western blot analysis of RPMVECs lysates was performed using an anti‐rat HMGB1 antibody (Cell Signaling Technology, USA), and densitometric analysis was conducted using ImageJ software.

### 2.6 Statistical Analysis

Data were analyzed using GraphPad Prism 10 (GraphPad Software Inc.) and presented as mean ± SD. Normal distribution was assessed using the Kolmogorov–Smirnov test. Pairwise comparisons between experimental and control groups were made using a two‐tailed paired or unpaired Student's *t*‐test as appropriate. One‐way analysis of variance (ANOVA) was used for two‐group comparison of continuous variables with normal distribution, and two‐way ANOVA with appropriate post hoc tests was used for multiple‐group comparisons. A *p*‐value < 0.05 was considered statistically significant unless stated otherwise. Post hoc comparisons were performed using Tukey's multiple comparisons test.

## 3 Results

### 3.1 Right Ventricular Function in Rats Exposed to Methamphetamine

**FIGURE 1:**
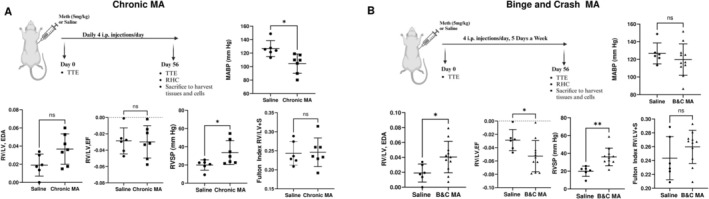
Effect of chronic methamphetamine (MA) and binge‐crash MA exposure on right ventricle (RV) function in rats. Male Wistar rats (220–280 g; *n* = 6–7 per group) received intraperitoneal injections of 5 mg/kg (+)‐methamphetamine (MA) dissolved in saline. Two dosing paradigms were used. (A) Chronic regimen: MA administered four times daily at 6 h intervals, seven days a week, for eight weeks. (B) Binge‐crash (B&C) regimen: MA given four times daily at 2 h intervals for five consecutive days each week, followed by two drug‐free days, repeated for eight weeks. Echocardiography and hemodynamic analysis included mean blood pressure, ∆RV/LV end‐diastolic area (EDA), ∆RV/LV ejection fraction (EF), RVSP, and Fulton index. *p < 0.05, **p < 0.01, versus saline control.

When comparing chronic and binge‐crash MA treatment for 8 weeks, no significant differences were observed in ∆RV/LV end‐diastolic area (EDA) or ∆RV/LV ejection fraction (EF) between chronic MA‐treated rats and saline controls. Chronic MA administration resulted in a significant reduction in mean arterial pressure (MAP) when compared with the saline group as shown in Figure 1A. Despite this decrease, RV systolic pressure (RVSP) was significantly elevated while the Fulton index remained unchanged, indicating increased RV afterload without detectable RV hypertrophy.

In contrast, rats subjected to binge‐crash MA regimen exhibited marked RV abnormalities. These animals showed significantly increased RV/LV EDA and reduced RV/LV EF compared with saline‐treated controls without any differences in mean arterial pressures (Figure 1B). In addition, a statistically significant increase in RVSP accompanied by a trend toward increased Fulton index was observed. Collectively, binge‐crash MA exposure resulted in more pronounced RV dilation and dysfunction than chronic MA exposure.

RVSP measurements showed greater than 20% coefficient of variation (COV), reflecting inherent biological variability across animals. Notably, variability in binge‐crash MA and saline groups was similar (27% COV in both groups), whereas the Chronic MA group demonstrated greater variability (38% COV). For the Fulton index, variability was lowest in the binge‐crash group (9% COV) and higher in the chronic MA (15% COV) group relative to the saline group (12% COV). Overall, this data suggests that the binge‐crash model exhibits less variability in RV dysfunction, supporting its consistency as a PH model.

### 3.2 Pulmonary Vascular Changes in Rats Exposed to Chronic or Binge‐Crash Methamphetamine

**FIGURE 2:**
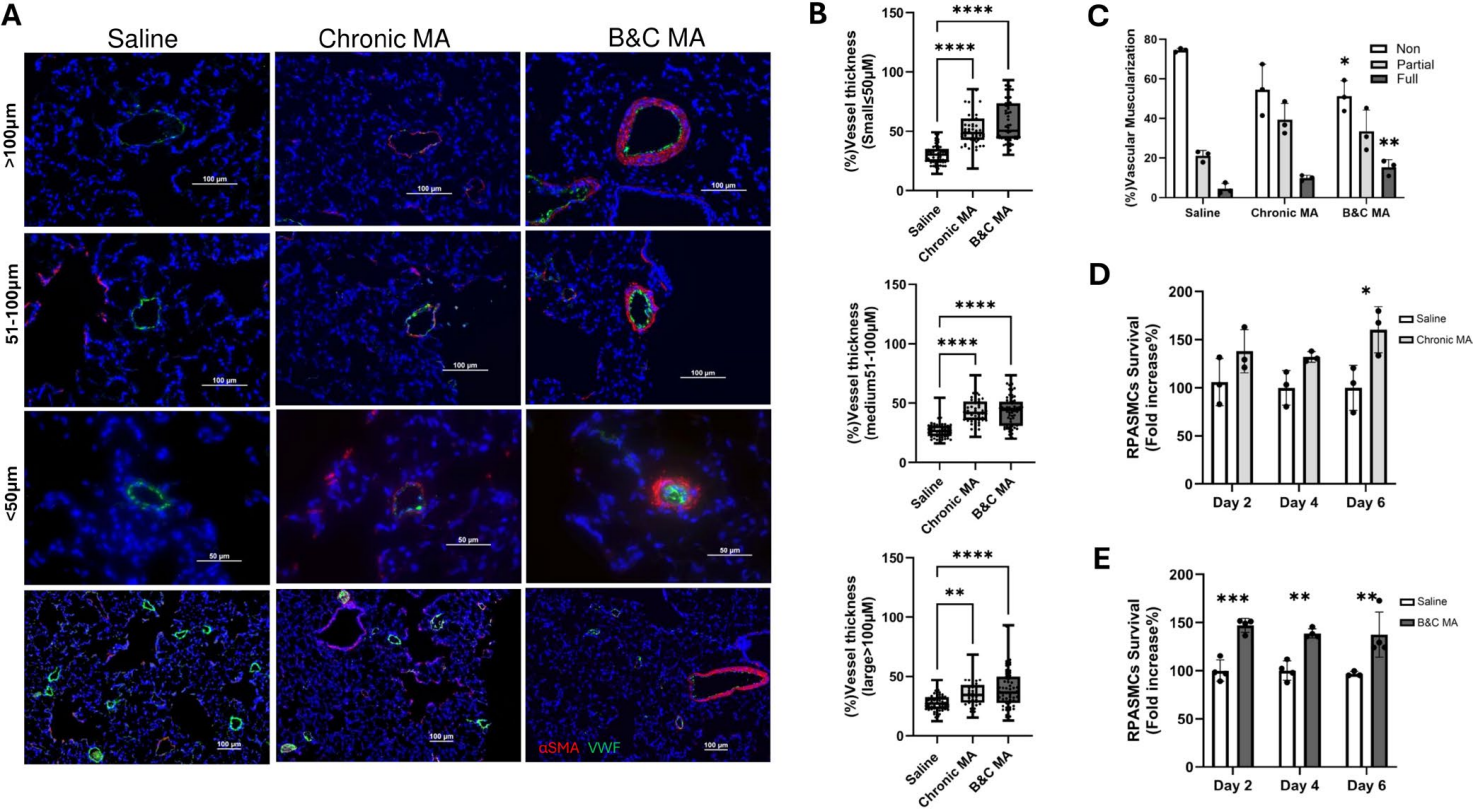
(A) Representative lung sections stained for α‐smooth muscle Actin (α‐SMA, red) and von Willebrand factor (vWF, green) from rats treated with chronic MA, binge‐crash (B&C) MA, or saline control. (B) Quantification of vessel thickness in rats treated with or without Chronic or B&C MA. Approximately 60 large, medium, or small vessels were measured from randomly selected *N* = 4–7 animals per group. Measurements were performed on pulmonary arteries identified in α‐SMA‐stained lung sections, and the average wall thickness was calculated for each animal. (C) Percentage muscularization of ≤ 50 μm distal vessels in saline, chronic, and B&C rats. α‐SMA and vWF immunofluorescent‐stained lung sections from 3 animals per group were categorized based on the presence or absence of smooth muscle coverage. (D, E) Proliferation analysis of pulmonary arterial smooth muscle cells isolated from chronic and B&C MA rats (*N* = 3–4 animals per group). Cells were cultured without serum and growth factor for 2, 4, or 6 days and assayed using the MTS assay. *p < 0.05, **p < 0.01, ***p < 0.001, ****p < 0.0001 versus saline control.

Histological evaluation of lung sections using vWF and α‐SMA immunostaining demonstrated medial hypertrophy of pulmonary arteries in both chronic and binge‐crash MA‐treated rats, associated with smooth muscle hyperplasia as shown in the representative images in Figure 2A. Quantitative morphometric analysis of α‐SMA‐stained vessels showed increased wall thickness across all vessel size categories (< 50 μm, 50–100 μm, and > 100 μm) in both MA‐treated groups (Figure 2B). Notably, lungs from binge‐crash MA rats displayed a reduced number of distal vessels but a significantly higher proportion of muscularized distal vessels (Figure 2C) as well as sporadic endothelial proliferation within partially obstructed small arteries (Figure 2A). Consistent with these findings, pulmonary arterial smooth muscle cells isolated from both chronic and binge‐crash MA rats demonstrated increased proliferation as measured by MTS assay (Figure 2D,E). Although smooth muscle hyperplasia occurred in both models, muscularization and rarefaction of distal arteries were more prominent in the binge‐crash group.

### 3.3 Increased HMGB1 Levels in Plasma and Pulmonary Microvascular Endothelial Cells After MA Exposure

**FIGURE 3:**
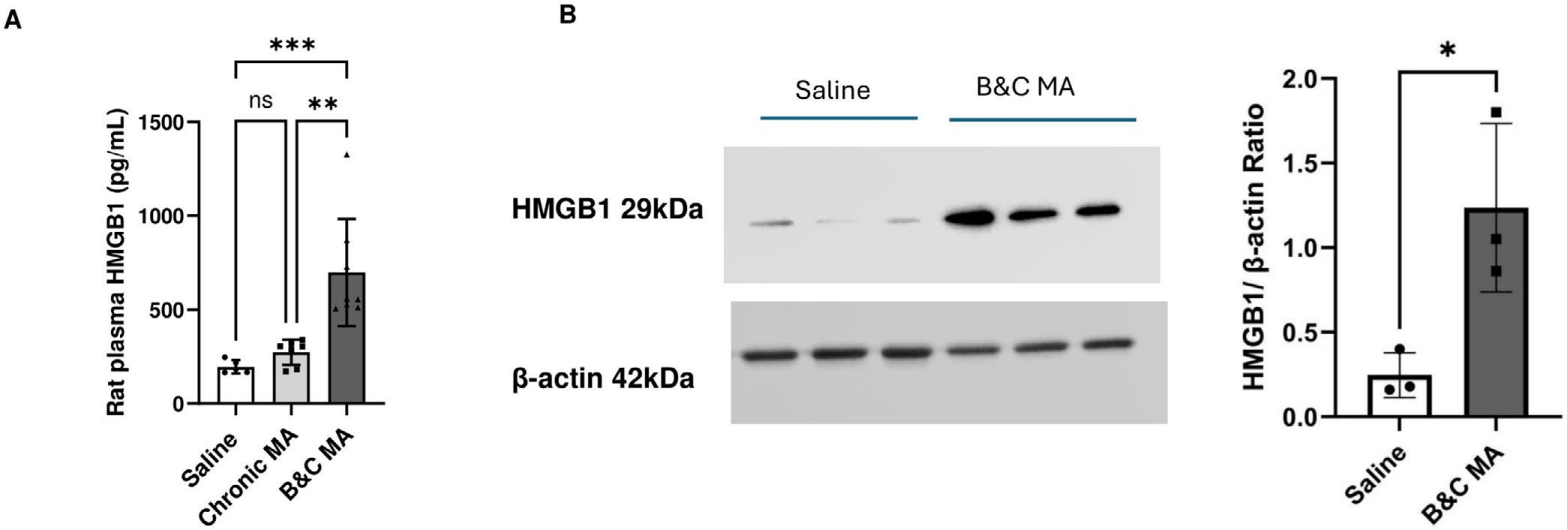
(A) HMGB1 concentrations in the plasma from rats treated with chronic MA, binge‐crash (B&C) MA, and saline (*n* = 5–8/group) measured by ELISA. (B) Western blot analysis of HMGB1 protein in pulmonary microvascular endothelial cells isolated from B&C MA and saline‐treated rats. *p < 0.05, **p < 0.01, ***p < 0.001.

Given prior evidence implicating HMGB1 in PAH pathogenesis (Zemskova et al. 2020; Dai et al. 2019), we assessed its levels following MA exposure. Plasma HMGB1 concentrations were significantly higher in binge‐crash MA‐treated rats when compared with saline controls (Figure 3A). Moreover, the rise in plasma HMGB1 in these rats was also significantly greater when compared with the chronic MA group. Western blot analysis of PMVECs from binge‐crash MA rats further confirmed higher expression of HMGB1, as shown in Figure 3B, whereas no significant changes were observed in PASMCs from these rats (data not shown).

### 3.4 HMGB1 Released From MA‐Treated Endothelial Cells Promotes Smooth Muscle Proliferation

**FIGURE 4:**
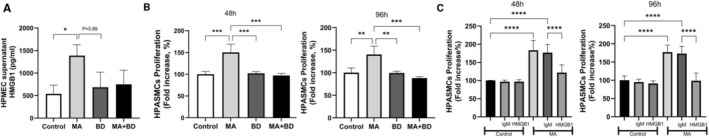
(A) HMGB1 levels assessed using ELISA in the supernatant of pulmonary microvascular endothelial cells (HPMECs) treated with MA in the presence or absence of sigma‐1 receptor antagonist: BD‐1047 (10 nM). (B) Proliferation analysis of human pulmonary arterial smooth muscle cells (HPASMCs) after exposure to supernatants from BD‐1407 ± MA‐treated HPMECs, assessed using CyQuant proliferation assay. (C) HPASMC proliferation after exposure to MA‐treated HPMEC supernatants, in the absence and presence of an HMGB1‐neutralizing antibody. Cells were cultured under serum‐free conditions for 2 or 4 days and assayed using CyQuant. *p < 0.05, **p < 0.01, ***p < 0.001, ****p < 0.0001. The data represent the average of 2–3 independent experiments; each performed in triplicate.

Previous studies have demonstrated that MA acts through sigma 1/2 receptors located in the endoplasmic reticulum (Yasui and Su 2016). We also previously reported the presence of sigma‐1 receptor (Sig‐1R) in pulmonary endothelial cells, whereas expression of the dopamine receptor, another known MA binding receptor, was minimal (Dalvi et al. 2014). Therefore, we tested whether the MA induces an increase in HMGB1 expression in human pulmonary microvascular endothelial cells (HPMECs) and whether this effect is dependent on Sig‐1R signaling. MA treatment significantly increased HMGB1 secretion from HPMECs compared with the control group, and pretreatment of cells with Sig‐1R antagonist BD‐1047 attenuated this effect (Figure 4A). Correspondingly, transfer of conditioned media from MA‐treated HPMECs to human pulmonary arterial smooth muscle cells (HPASMCs) resulted in smooth muscle hyperplasia (Figure 4B). This proliferative effect was abolished when HPMECs were pretreated with BD‐1047, indicating that Sig‐1R‐dependent HMGB1 release in response to MA contributes to smooth muscle proliferation. Additionally, HPASMC proliferation induced by MA‐HPMEC supernatant was prevented by co‐treatment with HMGB1‐neutralizing antibody (Figure 4C), further confirming that endothelial‐derived HMGB1 is one of the mediators of MA‐induced smooth muscle hyperplasia.

## 4 Discussion

Methamphetamine‐associated pulmonary arterial hypertension is an increasingly recognized and severe form of drug‐induced pulmonary vascular disease, yet the mechanisms linking patterns of MA exposure to pulmonary vascular remodeling and RV dysfunction remain incompletely defined (Charoenpong et al. 2023; Cruickshank and Dyer 2009). In this study, we demonstrate that binge‐crash MA exposure in rats induces substantially more severe RV dysfunction than chronic MA exposure, despite both regimens producing marked pulmonary vascular remodeling with smooth muscle hyperplasia. Notably, binge‐crash exposure was uniquely associated with muscularization and greater rarefaction of distal pulmonary vessels and a significant increase in HMGB1 levels in both plasma and pulmonary microvascular endothelial cells. The Sig‐1R–dependent release of HMGB1 from MA‐exposed HPMECs further promoted smooth muscle hyperplasia, suggesting a contributory role for HMGB1 in MA‐mediated pulmonary vascular remodeling.

A connection between MA misuse and PAH was initially suggested in 1993 with the first case report of PAH in MA users (Schaiberger et al. 1993). Since then, multiple epidemiologic studies have strengthened this association, and MA misuse is now classified as a definite cause of PAH (Zamanian et al. 2018; Kolaitis et al. 2021) (Ramirez 3rd et al. 2018; Charoenpong et al. 2023; O'Neill et al. 2025). Among the systemic consequences of MA abuse, PAH is one of the most morbid complications, associated with progressive RV failure and increased mortality. PET imaging studies using radiolabeled MA have shown rapid and preferential accumulation of MA in lung tissue, highlighting the vulnerability of the pulmonary vasculature to MA‐induced toxicity (Volkow et al. 2010). Suggested mechanisms include oxidative stress, endothelial dysfunction, inflammatory activation, and disruption of the alveolar–capillary interface. Yet, the molecular pathways that link MA exposure to PAH pathogenesis remain poorly understood, in part due to the absence of animal models that reliably reproduce the hemodynamic features of MA‐PAH.

Patterns of human MA intake are heterogeneous, but epidemiological studies show that chronic users typically consume MA on approximately 6 days per week, whereas binge users engage in intense use over 4 or more consecutive days, followed by abstinence for several days (Simon et al. 2002; Cho and Melega 2002) Although self‐reported exposure patterns are imperfect, these data highlight that human MA use generally involves repeated, high‐dose exposure over extended periods, which is a critical feature to capture in preclinical modeling of MAPAH.

Previous attempts to model MA‐induced pulmonary hypertension in rodents have not been successful in yielding hemodynamic outcomes (Liang et al. 2020; Liu et al. 2013). A major translational challenge is the marked species difference in MA pharmacokinetics between humans and rodents (Milesi‐Hallé et al. 2015; Milesi‐Hallé et al. 2005; Cho et al. 2001). Rodents metabolize MA far more rapidly than humans, with a rodent half‐life of ~1–2 h compared to ~10–12 h in humans. Consequently, rodents require higher mg/kg doses to achieve plasma and tissue exposures that approximate the cumulative burden experienced by chronic human users.

Rat studies using higher and prolonged dose regimens (5–10 mg/kg once or twice daily for 5–8 weeks) demonstrated pulmonary vascular remodeling, mitochondrial injury, oxidative stress, and inflammation (Liu et al. 2013; Wang et al. 2017b), but did not show increased RV systolic pressure (RVSP). Mouse studies commonly employed lower dosing (0.5 mg/kg) (Labazi et al. 2021) even though metabolism in mice is higher than in rats (MacAvoy et al. 2006; Kowalski and Bruce 2014). Mice showed less reproducible pulmonary vascular changes, but did reveal MA‐induced right ventricular hypertrophic gene programs and vascular smooth muscle cell phenotype transitions (Labazi et al. 2021). Similarly, short‐term amphetamine exposure (10 mg/kg twice daily for 3 days) followed by 4 days of hypoxia failed to elevate RVSP, even though significant endothelial mitochondrial dysfunction and DNA damage were observed (Chen et al. 2017).

To develop a preclinical MA‐PAH model that reflects human‐relevant drug‐use behaviors, our initial attempt using a 20 mg/kg dose resulted in significant mortality within four days. We therefore implemented a step‐down titration (10 mg/kg followed by 5 mg/kg) to reduce acute toxicity while maintaining sustained exposure. After multiple trials, we determined that 5 mg/kg daily provided the optimal balance, as animals survived the full 8‐week protocol and exhibited progressive weight loss consistent with chronic MA toxicity. We limited this study to male Wistar rats because previous pre‐clinical PH studies consistently report a more severe phenotype in males (Kwan et al. 2024; Rafikova et al. 2015; Frump et al. 2015; Guihaire et al. 2015). Additionally, our primary objective was to compare chronic and binge‐crash MA dosage regimens; we selected male rats to minimize biological variability. Sex differences in the MA pharmacokinetics have been documented in rats, with females showing lower clearance and exhibiting higher than expected METH concentration in serum after multiple MA administrations (Milesi‐Hallé et al. 2005, 2015). Overall, pharmacokinetic variability between male and female rats suggests that female rats may require distinct dosing strategies to model MA‐PAH. As next steps, future studies are warranted to optimize and validate MA‐PAH in female rats to directly examine the sex dependent differences in the disease development.

A central finding of our study is that the pattern of MA exposure, rather than cumulative dose alone, critically determines cardiopulmonary outcomes in preclinical animal models. Chronic MA exposure resulted in reduced mean arterial pressure and increased RVSP, while the RV ejection fraction and the Fulton index remained unchanged, suggesting a compensated RV adaptation to increased afterload. In contrast, binge‐crash MA exposure led to RV dilation, reduced RV ejection fraction, elevated RVSP, and moderate RV hypertrophy, indicating maladaptive RV remodeling.

The binge‐crash model demonstrated more severe pulmonary vascular remodeling, including increased distal muscularization, endothelial proliferation, and vascular rarefaction, and this may have contributed to a substantial rise in pulmonary vascular resistance and chronic RV pressure afterload. Our results support the concept that episodic surges of MA during binge‐crash use overwhelm adaptive RV responses, leading to RV‐pulmonary vascular uncoupling. Recent mouse studies using binge‐crash paradigms similarly reported elevated RVSP and pulmonary arterial medial thickening (Yasui and Su 2016), reinforcing the relevance of the exposure pattern.

The compensated RV phenotype in chronic MA exposure occurs alongside a paradoxical reduction in systemic MAP. MA normally enhances catecholamine signaling by blocking catecholamine transporters, reversing transporter flux, and inhibiting catecholamine‐metabolizing enzymes, thereby increasing dopamine and norepinephrine levels (Charoenpong et al. 2024). With prolonged exposure, however, sustained adrenergic stimulation likely leads to peripheral adrenergic receptor desensitization, norepinephrine depletion, and endothelial dysfunction. Together, these adaptations blunt vasopressor responsiveness, reduce systemic vascular resistance, and lower systemic MAP, even as the pulmonary vasculature continues to remodel under chronic MA exposure.

This systemic hypotension may modify RV loading conditions in ways that help preserve function. Reduced systemic arterial pressure decreases left ventricular (LV) afterload, lowering LV filling pressures and indirectly reducing pulmonary venous and left atrial pressures. This can partially mitigate RV wall stress despite elevated pulmonary arterial pressures. Lower systemic vascular resistance also reduces the energetic demands of the circulation, potentially preserving ventricular reserve. Furthermore, because the RV and LV share the interventricular septum and pericardial space, decreased LV afterload may help maintain septal contribution to RV systolic function, supporting RV‐pulmonary arterial coupling. Thus, the preserved RV ejection fraction in chronic MA exposure likely reflects a balance between increased pulmonary vascular resistance and the unloading effects of systemic hypotension.

Unlike the left ventricle, which is perfused predominantly during diastole, the right coronary artery perfuses the RV throughout both systole and diastole under normal conditions (Vogel‐Claussen et al. 2011; Gómez et al. 2001). Therefore, the reduction in MAP could impair the RV coronary perfusion. This places the RV in chronic MA‐exposed rats in a precarious position: sustaining function against elevated RV systolic pressure while myocardial oxygen supply is simultaneously threatened. The relatively modest pulmonary vascular remodeling in the chronic model, compared with the binge‐crash paradigm, may be what allows this balance to persist.

Beyond hemodynamic effects, MA can exert direct cardiotoxic effects that influence RV adaptation (Abdullah et al. 2020, 2022). In the chronic model, preserved RV ejection fraction and an unchanged Fulton index suggest that direct myocardial injury may not have yet progressed to the point of impairing global RV performance. In contrast, the binge‐crash MA exposure likely amplifies direct toxicity. Intermittent high‐dose exposure produces repeated surges in sympathetic activation and catecholamine‐mediated injury, leading to myocardial ischemia, oxidative stress, inflammation, fibrosis, and cardiomyocyte apoptosis (Abdullah et al. 2020). These experimental findings align with clinical observations demonstrating that patients with MA‐PAH, when matched to IPAH for mean pulmonary arterial pressures, exhibit worse right arterial and RV strain and more pronounced RV diastolic dysfunction compared to IPAH patients (Cruickshank and Dyer 2009).

Although the precise mechanisms through which MA promotes PAH are still evolving, proposed potential contributors include direct endothelial toxicity, oxidative stress, imbalances in vasoactive mediators, and reduced CES1 activity (Orcholski et al. 2017; Chen et al. 2017; Charoenpong et al. 2024). Our data show that both chronic and binge‐crash exposure increase pulmonary arterial smooth muscle proliferation; however, binge‐crash exposure uniquely resulted in worse endothelial injury characterized by focal endothelial proliferation and increased distal vessel rarefaction. Importantly, HMGB1 levels were significantly higher in binge‐crash rats compared with both saline and chronic MA groups.

HMGB1 is a well‐established DAMP implicated in PAH pathogenesis (Zemskova et al. 2020). Consistent with this, we observed that MA‐induced HMGB1 release from pulmonary microvascular endothelial cells was significantly attenuated by BD1047, a Sigma‐1 receptor antagonist. Prior studies have shown that MA binds Sig‐1R in endothelial cells and triggers intracellular stress signaling (Yasui and Su 2016). Our findings indicate that pulmonary endothelial cells are a primary source of MA‐induced HMGB1, and that release occurs via Sig‐1R‐dependent pathways. Conditioned media from MA‐treated endothelial cells increased smooth muscle cell proliferation in an HMGB1‐dependent manner, linking endothelial activation directly to smooth muscle hyperplasia. HMGB1 contributes to PAH progression through multiple pathways, including TLR4‐mediated inflammation, RAGE/NF‐κB–dependent smooth muscle proliferation, and ER‐stress–induced vascular remodeling (Qu et al. 2019; Wang et al. 2014; Zhang et al. 2023). Therapeutic HMGB1 blockade or inhibition of its downstream effectors has been shown to attenuate the development and progression of PAH in preclinical models (Goldenberg et al. 2019), highlighting its translational relevance.

In conclusion, binge‐crash MA exposure induces profound RV dysfunction surpassing the effects of chronic MA dosing. These pathological changes are closely associated with elevated HMGB1 levels and Sig‐1R‐dependent endothelial activation that drives smooth muscle proliferation and may also reflect a direct effect of HMGB1 on the right heart. Collectively, our findings establish a physiologically relevant preclinical rat model of MA‐PAH and suggest HMGB1 signaling as a potential contributor and therapeutic target that warrants further in vivo validation, connecting MA exposure patterns to pulmonary vascular injury.

## Author Contributions

Ashok Kumar and Ling Chen performed animal experiments. Cell‐culture experiments were performed by Balaji Krishnamachary and Aatish Mahajan. Ashok Kumar, Aatish Mahajan, Balaji Krishnamachary, Ling Chen, and Navneet K. Dhillon analyzed and interpreted the data. Ashok Kumar, Balaji Krishnamachary, Aatish Mahajan, and Navneet K. Dhillon contributed to writing the manuscript. Navneet K. Dhillon designed, conceptualized, and supervised the research. All authors read the manuscript and approved the study.

## Funding

The funds to carry out the study were provided by the National Institute of Health Grants R01 HL152832, R01 DA042715, and R01 HL129875 awarded to N.K.D.

## Conflicts of Interest

The authors declare no conflicts of interest.

## Data Availability

The data that support the findings of this study are available from the corresponding author upon reasonable request.
